# Does Video Gaming Have Impacts on the Brain: Evidence from a Systematic Review

**DOI:** 10.3390/brainsci9100251

**Published:** 2019-09-25

**Authors:** Denilson Brilliant T., Rui Nouchi, Ryuta Kawashima

**Affiliations:** 1 Department of Biomedicine, Indonesia International Institute for Life Sciences (i3L), East Jakarta 13210, Indonesia; 2 Smart Ageing Research Center (SARC), Tohoku University, Sendai 980-8575, Japan; rui@tohoku.ac.jp (R.N.); ryuta@tohoku.ac.jp (R.K.); 3 Department of Cognitive Health Science, Institute of Development, Aging and Cancer (IDAC), Tohoku University, Sendai 980-8575, Japan; 4 Department of Functional Brain Imaging, Institute of Development, Aging and Cancer (IDAC), Tohoku University, Sendai 980-8575, Japan

**Keywords:** brain, neuroplasticity, video gaming

## Abstract

Video gaming, the experience of playing electronic games, has shown several benefits for human health. Recently, numerous video gaming studies showed beneficial effects on cognition and the brain. A systematic review of video gaming has been published. However, the previous systematic review has several differences to this systematic review. This systematic review evaluates the beneficial effects of video gaming on neuroplasticity specifically on intervention studies. Literature research was conducted from randomized controlled trials in PubMed and Google Scholar published after 2000. A systematic review was written instead of a meta-analytic review because of variations among participants, video games, and outcomes. Nine scientific articles were eligible for the review. Overall, the eligible articles showed fair quality according to Delphi Criteria. Video gaming affects the brain structure and function depending on how the game is played. The game genres examined were 3D adventure, first-person shooting (FPS), puzzle, rhythm dance, and strategy. The total training durations were 16–90 h. Results of this systematic review demonstrated that video gaming can be beneficial to the brain. However, the beneficial effects vary among video game types.

## 1. Introduction

Video gaming refers to the experience of playing electronic games, which vary from action to passive games, presenting a player with physical and mental challenges. The motivation to play video games might derive from the experience of autonomy or competing with others, which can explain why video gaming is pleasurable and addictive [1].

Video games can act as “teachers” depending on the game purpose [2]. Video gaming has varying effects depending on the game genre. For instance, an active video game can improve physical fitness [3,4,5,6], whereas social video games can improve social behavior [7,8,9]. The most interesting results show that playing video games can change cognition and the brain [10,11,12,13].

Earlier studies have demonstrated that playing video games can benefit cognition. Cross-sectional and longitudinal studies have demonstrated that the experience of video gaming is associated with better cognitive function, specifically in terms of visual attention and short-term memory [14], reaction time [15], and working memory [16]. Additionally, some randomized controlled studies show positive effects of video gaming interventions on cognition [17,18]. Recent meta-analytical studies have also supported the positive effects of video gaming on cognition [10,11,12,13]. These studies demonstrate that playing video games does provide cognitive benefits.

The effects of video gaming intervention are ever more widely discussed among scientists [13]. A review of the results and methodological quality of recently published intervention studies must be done. One systematic review of video gaming and neural correlates has been reported [19]. However, the technique of neuroimaging of the reviewed studies was not specific. This systematic review reviewed only magnetic resonance imaging (MRI) studies in contrast to the previous systematic review to focus on neuroplasticity effect. Neuroplasticity is capability of the brain that accommodates adaptation for learning, memorizing, and recovery purposes [19]. In normal adaptation, the brain is adapting to learn, remember, forget, and repair itself. Recent studies using MRI for brain imaging techniques have demonstrated neuroplasticity effects after an intervention, which include cognitive, exercise, and music training on the grey matter [20,21,22,23,24] and white matter [25,26,27,28,29]. However, the molecular mechanisms of the grey and white matter change remain inconclusive. The proposed mechanisms for the grey matter change are neurogenesis, gliogenesis, synaptogenesis, and angiogenesis, whereas those for white matter change are myelin modeling and formation, fiber organization, and angiogenesis [30]. Recent studies using MRI technique for brain imaging have demonstrated video gaming effects on neuroplasticity. Earlier imaging studies using cross-sectional and longitudinal methods have shown that playing video games affects the brain structure by changing the grey matter [31,32,33], white matter [34,35], and functional connectivity [36,37,38,39]. Additionally, a few intervention studies have demonstrated that playing video games changed brain structure and functions [40,41,42,43].

The earlier review also found a link between neural correlates of video gaming and cognitive function [19]. However, that review used both experimental and correlational studies and included non-healthy participants, which contrasts to this review. The differences between this and the previous review are presented in Table 1. This review assesses only experimental studies conducted of healthy participants. Additionally, the cross-sectional and longitudinal studies merely showed an association between video gaming experiences and the brain, showing direct effects of playing video games in the brain is difficult. Therefore, this systematic review specifically examined intervention studies. This review is more specific as it reviews intervention and MRI studies on healthy participants. The purposes of this systematic review are therefore to evaluate the beneficial effects of video gaming and to assess the methodological quality of recent video gaming intervention studies. 

## 2. Materials and Methods

### 2.1. Search Strategy

This systematic review was designed in accordance with the PRISMA checklist [44] shown in Appendix Table A1. A literature search was conducted using PubMed and Google Scholar to identify relevant studies. The keywords used for the literature search were combinations of “video game”, “video gaming”, “game”, “action video game”, “video game training”, “training”, “play”, “playing”, “MRI”, “cognitive”, “cognition”, “executive function”, and “randomized control trial”.

### 2.2. Inclusion and Exclusion Criteria

The primary inclusion criteria were randomized controlled trial study, video game interaction, and MRI/fMRI analysis. Studies that qualified with only one or two primary inclusions were not included. Review papers and experimental protocols were also not included. The secondary inclusion criteria were publishing after 2000 and published in English. Excluded were duration of less than 4 weeks or unspecified length intervention or combination intervention. Also excluded were studies of cognition-based games, and studies of participants with psychiatric, cognitive, neurological, and medical disorders.

### 2.3. Quality Assessment

Each of the quality studies was assessed using Delphi criteria [45] with several additional elements [46]: details of allocation methods, adequate descriptions of control and training groups, statistical comparisons between control and training groups, and dropout reports. The respective total scores (max = 12) are shown in Table 3. The quality assessment also includes assessment for risk of bias, which is shown in criteria numbers 1, 2, 5, 6, 7, 9, and 12.

### 2.4. Statistical Analysis

Instead of a meta-analysis study, a systematic review of the video game training/video gaming and the effects was conducted because of the variation in ranges of participant age, video game genre, control type, MRI and statistical analysis, and training outcomes. Therefore, the quality, inclusion and exclusion, control, treatment, game title, participants, training period, and MRI analysis and specification of the studies were recorded for the respective games.

## 3. Results

The literature search made of the databases yielded 140 scientific articles. All scientific articles were screened based on inclusion and exclusion criteria. Of those 140 scientific articles, nine were eligible for the review [40,41,42,43,47,48,49,50,51]. Video gaming effects are listed in Table 2.

**Figure 1:**
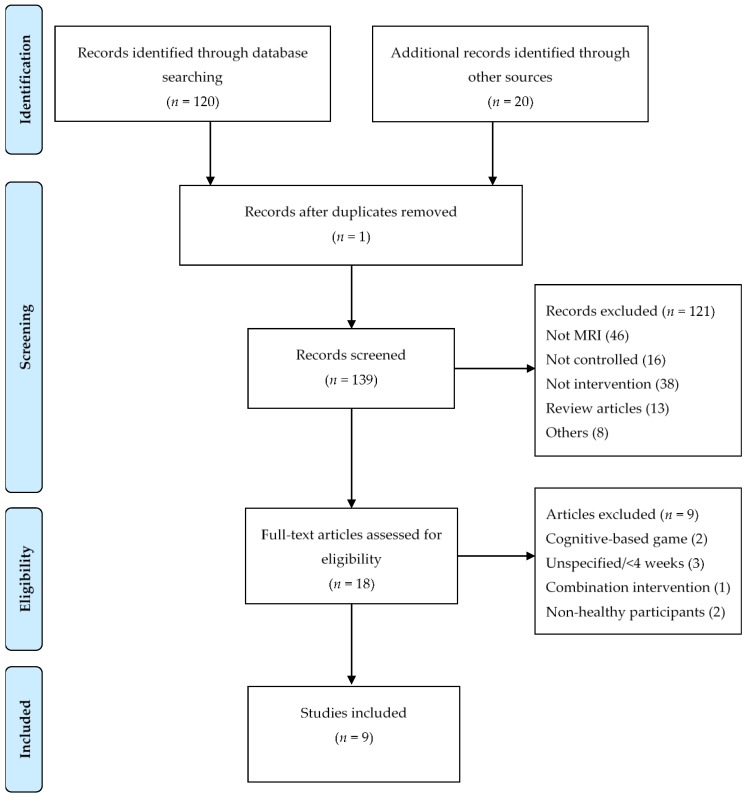
Flowchart of literature search.

We excluded 121 articles: 46 were not MRI studies, 16 were not controlled studies, 38 were not intervention studies, 13 were review articles, and eight were miscellaneous, including study protocols, non-video gaming studies, and non-brain studies. Of 18 included scientific articles, nine were excluded. Of those nine excluded articles, two were cognitive-based game studies, three were shorter than 4 weeks in duration or were without a specified length intervention, two studies used a non-healthy participant treatment, and one was a combination intervention study. A screening flowchart is portrayed in Figure 1.

### 3.1. Quality Assessment

The assessment methodology based on Delphi criteria [45] for the quality of eligible studies is presented in Table 3. The quality scores assigned to the studies were 3–9 (mean = 6.10; S.D. = 1.69). Overall, the studies showed fair methodological quality according to the Delphi criteria. The highest quality score of the nine eligible articles was assigned to “Playing Super Mario 64 increases hippocampal grey matter in older adult” published by West et al. in 2017, which scored 9 of 12. The scores assigned for criteria 6 (blinded care provider) and 7 (blinded patient) were lowest because of unspecified information related to blinding for those criteria. Additionally, criteria 2 (concealed allocation) and 5 (blinding assessor) were low because only two articles specified that information. All articles met criteria 3 and 4 adequately.

### 3.2. Inclusion and Exclusion

Most studies included participants with little or no experience with gaming and excluded participants with psychiatric/mental, neurological, and medical illness. Four studies specified handedness of the participants and excluded participants with game training experience. The inclusion and exclusion criteria are presented in Table 4.

### 3.3. Control Group

Nine eligible studies were categorized as three types based on the control type. Two studies used active control, five studies used passive control, and two studies used both active and passive control. A summary of the control group is presented in Table 5.

### 3.4. Game Title and Genre

Of the nine eligible studies, four used the same 3D adventure game with different game platforms, which were “Super Mario 64” original and the DS version. One study used first-person shooting (FPS) shooting games with many different game titles: “Call of Duty” is one title. Two studies used puzzle games: “Tetris” and “Professor Layton and The Pandora’s Box.” One study used a rhythm dance game: Dance Revolution. One study used a strategy game: “Space Fortress.” Game genres are presented in Table 6.

### 3.5. Participants and Sample Size

Among the nine studies, one study examined teenage participants, six studies included young adult participants, and two studies assessed older adult participants. Participant information is shown in Table 7. Numbers of participants were 20–75 participants (mean = 43.67; S.D. = 15.63). Three studies examined female-only participants, whereas six others used male and female participants. Six studies with female and male participants had more female than male participants.

### 3.6. Training Period and Intensity

The training period was 4–24 weeks (mean = 11.49; S.D. = 6.88). One study by Lee et al. had two length periods and total hours because the study examined video game training of two types. The total training hours were 16–90 h (mean = 40.63; S.D. = 26.22), whereas the training intensity was 1.5–10.68 h/week (mean = 4.96; S.D. = 3.00). One study did not specify total training hours. Two studies did not specify the training intensity. The training periods and intensities are in Table 8.

### 3.7. MRI Analysis and Specifications

Of nine eligible studies, one study used resting-state MRI analysis, three studies (excluding that by Haier et al. [40]) used structural MRI analysis, and five studies used task-based MRI analysis. A study by Haier et al. used MRI analyses of two types [40]. A summary of MRI analyses is presented in Table 9. The related resting-state, structural, and task-based MRI specifications are presented in Table 10, Table 11 and Table 12 respectively.

## 4. Discussion

This literature review evaluated the effect of noncognitive-based video game intervention on the cognitive function of healthy people. Comparison of studies is difficult because of the heterogeneities of participant ages, beneficial effects, and durations. Comparisons are limited to studies sharing factors.

### 4.1. Participant Age

Video gaming intervention affects all age categories except for the children category. The exception derives from a lack of intervention studies using children as participants. The underlying reason for this exception is that the brain is still developing until age 10–12 [52,53]. Among the eligible studies were a study investigating adolescents [40], six studies investigating young adults [41,42,43,47,49,51] and two studies investigating older adults [48,50].

Differences among study purposes underlie the differences in participant age categories. The study by Haier et al. was intended to study adolescents because the category shows the most potential brain changes. The human brain is more sensitive to synaptic reorganization during the adolescent period [54]. Generally, grey matter decreases whereas white matter increases during the adolescent period [55,56]. By contrast, the cortical surface of the brain increases despite reduction of grey matter [55,57]. Six studies were investigating young adults with the intention of studying brain changes after the brain reaches maturity. The human brain reaches maturity during the young adult period [58]. Two studies were investigating older adults with the intention of combating difficulties caused by aging. The human brain shrinks as age increases [56,59], which almost invariably leads to declining cognitive function [59,60].

### 4.2. Beneficial Effects

Three beneficial outcomes were observed using MRI method: grey matter change [40,42,50], brain activity change [40,43,47,48,49], and functional connectivity change [41]. The affected brain area corresponds to how the respective games were played.

Four studies of 3D video gaming showed effects on the structure of hippocampus, dorsolateral prefrontal cortex (DLPFC), cerebellum [42,43,50], and DLPFC [43] and ventral striatum activity [49]. In this case, the hippocampus is used for memory [61] and scene recognition [62], whereas the DLPFC and cerebellum are used for working memory function for information manipulation and problem-solving processes [63]. The grey matter of the corresponding brain region has been shown to increase during training [20,64]. The increased grey matter of the hippocampus, DLPFC, and cerebellum are associated with better performance in reference and working memory [64,65].

The reduced activity of DLPFC found in the study by Gleich et al. corresponds to studies that showed reduced brain activity associated with brain training [66,67,68,69]. Decreased activity of the DLPFC after training is associated with efficiency in divergent thinking [70]. 3D video gaming also preserved reward systems by protecting the activity of the ventral striatum [71].

Two studies of puzzle gaming showed effects on the structure of the visual–spatial processing area, activity of the frontal area, and functional connectivity change. The increased grey matter of the visual–spatial area and decreased activity of the frontal area are similar to training-associated grey matter increase [20,64] and activity decrease [66,67,68,69]. In this case, visual–spatial processing and frontal area are used constantly for spatial prediction and problem-solving of Tetris. Functional connectivity of the multimodal integration and the higher-order executive system in the puzzle solving-based gaming of Professor Layton game corresponds to studies which demonstrated training-associated functional connectivity change [72,73]. Good functional connectivity implies better performance [73].

Strategy gaming affects the DLPFC activity, whereas rhythm gaming affects the activity of visuospatial working memory, emotional, and attention area. FPS gaming affects the structure of the hippocampus and amygdala. Decreased DLPFC activity is similar to training-associated activity decrease [66,67,68,69]. A study by Roush demonstrated increased activity of visuospatial working memory, emotion, and attention area, which might occur because of exercise and gaming in the Dance Revolution game. Results suggest that positive activations indicate altered functional areas by complex exercise [48]. The increased grey matter of the hippocampus and amygdala are similar to the training-associated grey matter increase [20,64]. The hippocampus is used for 3D navigation purposes in the FPS world [61], whereas the amygdala is used to stay alert during gaming [74].

### 4.3. Duration

Change of the brain structure and function was observed after 16 h of video gaming. The total durations of video gaming were 16–90 h. However, the gaming intensity must be noted because the gaming intensity varied: 1.5–10.68 h per week. The different intensities might affect the change of cognitive function. Cognitive intervention studies demonstrated intensity effects on the cortical thickness of the brain [75,76]. A similar effect might be observed in video gaming studies. More studies must be conducted to resolve how the intensity can be expected to affect cognitive function.

### 4.4. Criteria

Almost all studies used inclusion criteria “little/no experience with video games.” The criterion was used to reduce the factor of gaming-related experience on the effects of video gaming. Some of the studies also used specific handedness and specific sex of participants to reduce the variation of brain effects. Expertise and sex are shown to affect brain activity and structure [77,78,79,80]. The exclusion criterion of “MRI contraindication” is used for participant safety for the MRI protocol, whereas exclusion criteria of “psychiatric/mental illness”, “neurological illness”, and “medical illness” are used to standardize the participants.

### 4.5. Limitations and Recommendations

Some concern might be raised about the quality of methodology, assessed using Delphi criteria [45]. The quality was 3–9 (mean = 6.10; S.D. = 1.69). Low quality in most papers resulted from unspecified information corresponding to the criteria. Quality improvements for the studies must be performed related to the low quality of methodology. Allocation concealment, assessor blinding, care provider blinding, participant blinding, intention-to-treat analysis, and allocation method details must be improved in future studies.

Another concern is blinding and control. This type of study differs from medical studies in which patients can be blinded easily. In studies of these types, the participants were tasked to do either training as an active control group or to do nothing as a passive control group. The participants can expect something from the task. The expectation might affect the outcomes of the studies [81,82,83]. Additionally, the waiting-list control group might overestimate the outcome of training [84].

Considering the sample size, which was 20–75 (mean = 43.67; S.D. = 15.63), the studies must be upscaled to emphasize video gaming effects. There are four phases of clinical trials that start from the early stage and small-scale phase 1 to late stage and large-scale phase 3 and end in post-marketing observation phase 4. These four phases are used for drug clinical trials, according to the food and drug administration (FDA) [85]. Phase 1 has the purpose of revealing the safety of treatment with around 20–100 participants. Phase 2 has the purpose of elucidating the efficacy of the treatment with up to several hundred participants. Phase 3 has the purpose of revealing both efficacy and safety among 300–3000 participants. The final phase 4 has the purpose of finding unprecedented adverse effects of treatment after marketing. However, because medical studies and video gaming intervention studies differ in terms of experimental methods, slight modifications can be done for adaptation to video gaming studies.

Several unresolved issues persist in relation to video gaming intervention. First, no studies assessed chronic/long-term video gaming. The participants might lose their motivation to play the same game over a long time, which might affect the study outcomes [86]. Second, meta-analyses could not be done because the game genres are heterogeneous. To ensure homogeneity of the study, stricter criteria must be set. However, this step would engender a third limitation. Third, randomized controlled trial video gaming studies that use MRI analysis are few. More studies must be conducted to assess the effects of video gaming. Fourth, the eligible studies lacked cognitive tests to validate the cognitive change effects for training. Studies of video gaming intervention should also include a cognitive test to ascertain the relation between cognitive function and brain change.

## 5. Conclusions

The systematic review has several conclusions related to beneficial effects of noncognitive-based video games. First, noncognitive-based video gaming can be used in all age categories as a means to improve the brain. However, effects on children remain unclear. Second, noncognitive-based video gaming affects both structural and functional aspects of the brain. Third, video gaming effects were observed after a minimum of 16 h of training. Fourth, some methodology criteria must be improved for better methodological quality. In conclusion, acute video gaming of a minimum of 16 h is beneficial for brain function and structure. However, video gaming effects on the brain area vary depending on the video game type.

## Figures and Tables

**Table 1:** Differences between previous review and current review.

Difference	Previous Review	Current Review
Type of reviewed studies	Experimental and correlational studies	Experimental studies only
Neuroimaging technique of reviewed studies	CT, fMRI, MEG, MRI, PET, SPECT, tDCS, EEG, and NIRS	fMRI and MRI only
Participants of reviewed studies	Healthy and addicted participant	Healthy participants Only

CT, computed tomography; fMRI, functional magnetic resonance imaging; MEG, magnetoencephalography MRI, magnetic resonance imaging; PET, positron emission tomography; SPECT, single photon emission computed tomography; tDCS, transcranial direct current stimulation; EEG, electroencephalography; NIRS, near-infrared spectroscopy.

**Table 2:** Summary of beneficial effect of video gaming.

Author	Year	Participant Age	Game Genre	Control	Duration	Beneficial Effect
Gleich et al. [43]	2017	18–36	3D adventure	passive	8 weeks	Increased activity in hippocampus
						Decreased activity in DLPFC
Haier et al. [40]	2009	12–15	puzzle	passive	3 months	Increased GM in several visual–spatial processing area
						Decreased activity in frontal area
Kuhn et al. [42]	2014	19–29	3D adventure	passive	8 weeks	Increased GM in hippocampal, DLPFC and cerebellum
Lee et al. [47]	2012	18–30	strategy	active	8–10 weeks	Decreased activity in DLPFC
					8–11 weeks	Non-significant activity difference
Lorenz et al. [49]	2015	19–27	3D adventure	passive	8 weeks	Preserved activity in ventral striatum
Martinez et al. [41]	2013	16–21	puzzle	passive	4 weeks	Functional connectivity change in multimodal integration system
						Functional connectivity change in higher-order executive processing
Roush [48]	2013	50–65	rhythm dance	active	24 weeks	Increased activity in visuospatial working memory area
						Increased activity in emotional and attention area
				passive		Similar compared to active control-
West et al. [50]	2017	55–75	3D adventure	active	24 weeks	Non-significant GM difference
				passive		Increased cognitive performance and short-term memory
						Increased GM in hippocampus and cerebellum
West et al. [51]	2018	18–29	FPS	active	8 weeks	Increased GM in hippocampus (spatial learner *)
						Increased GM in amygdala (response learner *)
						Decreased GM in hippocampus (response learner)

Duration was converted into weeks (1 month = 4 weeks); DLPFC, dorsolateral prefrontal cortex; GM, grey matter; FPS, first person shooting. * Participants were categorized based on how they played during the video gaming intervention.

**Table 3:** Methodological quality of eligible studies.

Author	Year	Q1	Q2	Q3	Q4	Q5	Q6	Q7	Q8	Q9	Q10	Q11	Q12	Score
Gleich et al. [43]	2017	1	0	1	1	0	0	0	0	0	1	1	1	6
Haier et al. [40]	2009	1	0	1	1	0	0	0	0	0	1	1	0	5
Kuhn et al. [42]	2014	1	0	1	1	0	0	0	0	0	1	1	0	5
Lee et al. [47]	2012	0	0	1	1	0	0	0	0	1	1	1	1	6
Lorenz et al. [49]	2015	1	0	1	1	0	0	0	1	0	1	1	1	7
Martinez et al. [41]	2013	0	0	1	1	0	0	0	0	0	0	1	0	3
Roush [48]	2013	1	1	1	1	1	0	0	0	1	1	0	0	7
West et al. [50]	2017	1	1	1	1	0	0	0	1	1	1	1	1	9
West et al. [51]	2018	0	0	1	1	1	0	0	1	1	1	0	1	7
Score		6	2	9	9	2	0	0	3	4	8	7	5	

Q1, Random allocation; Q2, Concealed allocation; Q3, Similar baselines among groups; Q4, Eligibility specified; Q5, Blinded assessor outcome; Q6, Blinded care provider; Q7, Blinded patient; Q8, Intention-to-treat analysis; Q9, Detail of allocation method; Q10, Adequate description of each group; Q11, Statistical comparison between groups; Q12, Dropout report (1, specified; 0, unspecified).

**Table 4:** Inclusion and exclusion criteria for eligible studies.

Author	Year	Inclusion			Exclusion				
		i1	i2	i3	e1	e2	e3	e4	e5
Gleich et al. [43]	2017	1	0	0	1	1	1	1	1
Haier et al. [40]	2009	1	0	1	1	1	1	0	0
Kuhn et al. [42]	2014	1	0	0	1	1	1	1	1
Lee et al. [47]	2012	1	1	0	1	1	0	1	0
Lorenz et al. [49]	2015	1	1	0	1	0	0	1	1
Martinez et al. [41]	2013	1	1	1	1	1	0	0	1
Roush [48]	2013	0	0	1	0	0	1	0	0
West et al. [50]	2017	1	1	0	1	1	1	1	0
West et al. [51]	2018	1	0	0	1	1	1	0	0
total		8	4	3	8	7	6	5	4

i1, Little/no experience in video gaming; i2, Right-handed; i3, Sex-specific; e1, Psychiatric/mental illness; e2, Neurological illness; e3, Medical illness; e4, MRI contraindication; e5, experience in game training.

**Table 5:** Control group examined eligible studies.

Control	Author	Year
Active control	Lee et al. [47]	2012
	West et al. [51]	2018
Passive control	Gleich et al. [43]	2017
	Haier et al. [40]	2009
	Kuhn et al. [42]	2014
	Lorenz et al. [49]	2015
	Martinez et al. [41]	2013
Active–passive control	Roush [48]	2013
	West et al. [50]	2017

**Table 6:** Genres and game titles of video gaming intervention.

Genre	Author	Year	Title
3D adventure	Gleich et al. [43]	2017	Super Mario 64 DS
	Kuhn et al. [42]	2014	Super Mario 64
	Lorenz et al. [49]	2015	Super Mario 64 DS
	West et al. [50]	2017	Super Mario 64
FPS	West et al. * [51]	2018	Call of Duty
Puzzle	Haier et al. [40]	2009	Tetris
	Martinez et al. [41]	2013	Professor Layton and The Pandora’s Box
Rhythm dance	Roush [48]	2013	Dance Revolution
Strategy	Lee et al. [47]	2012	Space Fortress

* West et al. used multiple games; other games are Call of Duty 2, 3, Black Ops, and World at War, Killzone 2 and 3, Battlefield 2, 3, and 4, Resistance 2 and Fall of Man, and Medal of Honor.

**Table 7:** Participant details of eligible studies.

Category	Author	Year	Age			Sample Size	Ratio (%)		Detail
			Lowest	Highest	Range		Female	Male	
Teenager	Haier et al. [40]	2009	12	15	3	44	70.45	29.54	Training (*n =* 24) Control (*n =* 20)
Young adult	Gleich et al. [43]	2017	18	36	18	26	100	0	Training (*n =* 15)
									Control (*n =* 11)
	Kuhn et al. [42]	2014	19	29	10	48	70.8	29.2	Training (*n =* 23)
									Control (*n =* 25)
	Lee et al. [47]	2012	18	30	12	75	61.4	38.6	Training A (*n =* 25)
									Training B (*n =* 25)
									Control (*n =* 25)
	Lorenz et al. [49]	2015	19	27	8	50	72	28	Training (*n =* 25
									Control (*n =* 25)
	Martinez et al. [41]	2013	16	21	5	20	100	0	Training (*n =* 10)
									Control (*n =* 10)
	West et al. [51]	2018	18	29	11	43	67.4	32.5	Action game (*n =* 21)
									Non-action game (*n =* 22)
Older adult	Roush [48]	2013	50	65	15	39	100	0	Training (*n =* 19)
									Active control (*n =* 15)
									Passive control (*n =* 5)
	West et al. [50]	2017	55	75	20	48	66.7	33.3	Training (*n =* 19)
									Active control (*n =* 14)
									Passive control (*n =* 15)

**Table 8:** Periods and intensities of video gaming intervention.

Author	Year	Length (Week)	Total Hours	Average Intensity (h/Week)
Gleich et al. [43]	2017	8	49.5	6.2
Haier et al. [40]	2009	12	18	1.5
Kuhn et al. [42]	2014	8	46.88	5.86
Lorenz et al. [49]	2012	8	28	3.5
Lee et al. [47]	2015	8–11 *	27	n/a
Martinez et al. [41]	2013	4	16	4
Roush [48]	2013	24	ns	n/a
West et al. [50]	2017	24	72	3
West et al. [51]	2018	8.4	90	10.68

The training length was converted into weeks (1 month = 4 weeks). ns, not specified; n/a, not available; * exact length is not available.

**Table 9:** MRI analysis details of eligible studies.

MRI Analysis	Author	Year	Contrast	Statistical Tool	Statistical Method	*p* Value
Resting	Martinez et al. [41]	2013	(post- > pre-training) > (post>pre-control)	MATLAB; SPM8	TFCE uncorrected	<0.005
Structural	Haier et al. * [40]	2009	(post>pre-training) > (post>pre-control)	MATLAB 7; SurfStat	FWE corrected	<0.005
	Kuhn et al. [42]	2014	(post>pre-training) > (post>pre-control)	VBM8; SPM8	FWE corrected	<0.001
	West et al. [50]	2017	(post>pre-training) > (post>pre-control)	Bpipe	Uncorrected	<0.0001
	West et al. [51]	2018	(post>pre-training) > (post>pre-control)	Bpipe	Bonferroni corrected	<0.001
Task	Gleich et al. [43]	2017	(post>pre-training) > (post>pre-control)	SPM12	Monte Carlo corrected	<0.05
	Haier et al. * [40]	2009	(post>pre-training) > (post>pre-control)	SPM7	FDR corrected	<0.05
	Lee et al. [47]	2012	(post>pre-training) > (post>pre-control)	FSL; FEAT	uncorrected	<0.01
	Lorenz et al. [49]	2015	(post>pre-training) > (post>pre-control)	SPM8	Monte Carlo corrected	<0.05
	Roush ^+^ [48]	2013	post>pre-training	MATLAB 7; SPM8	uncorrected	=0.001

* Haier et al. conducted structural and task analyses. + Compared pre-training and post-training between groups without using contrast. TFCE, Threshold Free Cluster Enhancement; FEW, familywise error rate; FDR, false discovery rate.

**Table 10:** Resting-State MRI specifications of eligible studies.

Author	Year	Resting State				Structural			
		Imaging	TR (s)	TE (ms)	Slice	Imaging	TR (s)	TE (ms)	Slice
**Martinez et al. [41]**	2013	gradient-echo planar image	3	28.1	36	T1-weighted	0.92	4.2	158

**Table 11:** Structural MRI specifications of eligible studies.

Author	Year	Imaging	TR (s)	TE (ms)
Kuhn et al. [42]	2014	3D T1 weighted MPRAGE	2.5	4.77
West et al. [50]	2017	3D gradient echo MPRAGE	2.3	2.91
West et al. [51]	2018	3D gradient echo MPRAGE	2.3	2.91

**Table 12:** Task-Based MRI specifications of eligible studies.

Author	Year	Task	BOLD				Structural			
			Imaging	TR (s)	TE (ms)	Slice	Imaging	TR (s)	TE (ms)	Slice
Gleich et al. [43]	2017	win–loss paradigm	T2 echo-planar image	2	30	36	T1-weighted	2.5	4.77	176
Haier et al. [40]	2009	Tetris	Functional echo planar	2	29	ns	5-echo MPRAGE	2.53	1.64; 3.5; 5.36; 7.22; 9.08	ns
Lee et al. [47]	2012	game control	fast echo-planar image	2	25	ns	T1-weighted MPRAGE	1.8	3.87	144
Lorenz et al. [49]	2015	slot machine paradigm	T2 echo-planar image	2	30	36	T1-weighted MPRAGE	2.5	4.77	ns
Roush [48]	2013	digit symbol substitution	fast echo-planar image	2	25	34	diffusion weighted image	ns	ns	ns

All analyses used 3 Tesla magnetic force; TR = repetition time; TE = echo time, ns = not specified.

## Appendix A

**Table A1:** PRISMA Checklist of the literature review.

Section/Topic	#	Checklist Item	Reported on Page #
**TITLE**			
Title	1	Identify the report as a systematic review, meta-analysis, or both.	1
**ABSTRACT**			
Structured summary	2	Provide a structured summary including, as applicable: background; objectives; data sources; study eligibility criteria, participants, and interventions; study appraisal and synthesis methods; results; limitations; conclusions and implications of key findings; systematic review registration number.	1
**INTRODUCTION**			
Rationale	3	Describe the rationale for the review in the context of what is already known.	1, 2
Objectives	4	Provide an explicit statement of questions being addressed related to participants, interventions, comparisons, outcomes, and study design (PICOS).	2
**METHODS**			
Protocol and registration	5	Indicate if a review protocol exists, if and where it is accessible (e.g., Web address), and if available, provide registration information including registration number.	2
Eligibility criteria	6	Specify study characteristics (e.g., PICOS, length of follow-up) and report characteristics (e.g., years considered, language, publication status) used as criteria for eligibility, giving rationale.	2
Information sources	7	Describe all information sources (e.g., databases with dates of coverage, contact with study authors to identify additional studies) in the search and date last searched.	2
Search	8	Present full electronic search strategy for at least one database, including any limits used, such that it could be repeated.	2
Study selection	9	State the process for selecting studies (i.e., screening, eligibility, included in systematic review, and if applicable, included in the meta-analysis).	3
Data collection process	10	Describe method of data extraction from reports (e.g., piloted forms, independently, in duplicate) and any processes for obtaining and confirming data from investigators.	3
Data items	11	List and define all variables for which data were sought (e.g., PICOS, funding sources) and any assumptions and simplifications made.	3
Risk of bias in individual studies	12	Describe methods used for assessing risk of bias of individual studies (including specification of whether this was done at the study or outcome level), and how this information is to be used in any data synthesis.	2
Summary measures	13	State the principal summary measures (e.g., risk ratio, difference in means).	-
Synthesis of results	14	Describe the methods of handling data and combining results of studies, if done, including measures of consistency (e.g., I^2^) for each meta-analysis.	-
Risk of bias across studies	15	Specify any assessment of risk of bias that might affect the cumulative evidence (e.g., publication bias, selective reporting within studies).	-
Additional analyses	16	Describe methods of additional analyses (e.g., sensitivity or subgroup analyses, meta-regression), if done, indicating which were pre-specified.	-
**RESULTS**			
Study selection	17	Give numbers of studies screened, assessed for eligibility, and included in the review, with reasons for exclusions at each stage, ideally with a flow diagram.	3,5
Study characteristics	18	For each study, present characteristics for which data were extracted (e.g., study size, PICOS, follow-up period) and provide the citations.	5-11
Risk of bias within studies	19	Present data on risk of bias of each study, and if available, any outcome level assessment (see item 12).	5,6
Results of individual studies	20	For all outcomes considered (benefits or harms), present, for each study: (a) simple summary data for each intervention group (b) effect estimates and confidence intervals, ideally with a forest plot.	4
Synthesis of results	21	Present results of each meta-analysis done, including confidence intervals and measures of consistency.	-
Risk of bias across studies	22	Present results of any assessment of risk of bias across studies (see Item 15).	-
Additional analysis	23	Give results of additional analyses, if done (e.g., sensitivity or subgroup analyses, meta-regression [see Item 16]).	-
**DISCUSSION**			
Summary of evidence	24	Summarize the main findings including the strength of evidence for each main outcome; consider their relevance to key groups (e.g., healthcare providers, users, and policy makers).	12,13
Limitations	25	Discuss limitations at study and outcome level (e.g., risk of bias), and at review-level (e.g., incomplete retrieval of identified research, reporting bias).	13
Conclusions	26	Provide a general interpretation of the results in the context of other evidence, and implications for future research.	14
**FUNDING**			
Funding	27	Describe sources of funding for the systematic review and other support (e.g., supply of data); role of funders for the systematic review.	14

For more information, visit: www.prisma-statement.org.
